# Dissertation on the Perforating Ulcer of the Stomach, &c.

**Published:** 1842-04

**Authors:** 


Art. II.—*Dissertationis de Ulcere Ventriculi Perforante*, Particula Prior, Quam pro licentiâ, &c. Eduardus A. Dahlerup.—*Havniæ*, 1840. 12mo, pp. 77.

*Dissertation on the Perforating Ulcer of the Stomach.* Part I. By Edward A. Dahlerup.—*Copenhagen*, 1840.

We have here a very well-digested and complete dissertation on the morbid anatomy of this interesting disease of the stomach, of which the author has added eighteen cases to those scattered through the various works of Baillie, Cruveilhier, Abercrombie, Rokitanski, and others. Few diseases have more constant characters. The patient is generally one who has till within a short time of the fatal seizure been deemed thoroughly healthy, or who has only suffered from slight dyspepsia. But suddenly the well-known signs of acute peritonitis from perforation come on, and in the space of from one to four or five days terminate fatally. After death the disease is found to have consisted of an ulcer of the stomach, round or oval in form, from one fourth to one half of an inch in its greatest diameter, with smooth clean-cut edges, as if a portion of the membrane had been *punched* out, and which has gradually made its way from within outwards, till, penetrating the peritoneal coat, or causing it to slough, it has permitted the fatal effusion. In general there is no other disease of the stomach except a slight thickening and induration of the membranes immediately surrounding the ulcer, with which there is also not unfrequently associated such adhesion of the stomach to the adjacent viscera, as for a time or permanently prevents the effusion from taking place.

Such are its general characters; the summary of the author affords some interesting results from the comparison of the numerous cases which he has collated, in regard to the less obvious features of the disease. With respect to the situation of the ulcers, they are almost invariably seated in or near the lesser arch, (in 79 cases Rokitanski found but one with such an ulcer in the fundus,) they are rather more frequent on the posterior than on the anterior surface, and most commonly lie nearer to the cardia than to the pylorus. Much more rarely ulcers of similar form and characters are found in the duodenum. In the great majority of cases they occur singly: in some (in 12 for example of the 79 examined by Rokitanski) there were two, in a few three, and in yet fewer more than three. They rarely equal, and still more rarely surpass, an inch in their greatest diameter; and very rarely deviate from the regular circular or oval form, except the ulcer have by some accident been enabled to spread more widely than usual before perforating. The thickening and hardness which are generally found surrounding them result from deposition in the cellular coats of the stomach, the muscular fibres being commonly traceable unaltered to the very margin of the ulcer, and this hardening is effected by the addition of a homogeneous uniform substance altogether different from that found in carcinoma. The margins of the perforation are almost always even and abrupt, or gradually and smoothly shelving from within outwards: they are very rarely softened or discoloured, but sometimes exhibit an appearance as if the ulcer had made progress in definite stages, destroying one coat, then staying, then destroying a less extent of the next coat, and so on. Before perforation the base of the ulcer is generally firm and smooth, more rarely rugged, vascular, and softened; when it reaches the peritoneum it causes it to slough, and sometimes when the perforation takes place, the slough not being swept clean away, is found adhering to one of the margins of the ulcer.

The cause, the origin, and the early progress of this disease, are almost unknown. Its termination is in the large majority of cases fatal, by the effusion into the peritoneum, but there is sufficient evidence that in some instances the ulcers have cicatrized. Such was probably the case of Beclard himself. Cruveilhier and Rokitanski have often found cicatrices in the stomach, of such a form as could leave little doubt they had been preceded by ulcers of this kind.

We have ourselves found such cicatrices in three instances. They presented all the ordinary characters of the cicatrix of a deep circular ulcer of the skin; and, indeed, the whole early progress of this disease is exactly analogous to that of the ordinary circular deep ulcer of the extremities. It derives all its importance from its position, which is such, that after having made a certain extent of progress, it leads to an accident, the effusion of the contents of the stomach, which brings about a result such as the ulceration alone would perhaps never have induced.

The disease has considerable interest in a medico-legal point of view. In two cases that we examined there was some suspicion of poison having been administered, for the patients were destroyed within twenty-four hours of having eaten hearty meals, by an illness which commenced shortly after they had finished eating. The form of the ulcer, the firmness of its borders, the evenness of its edges, the healthiness and absence of inflammation in the rest of the stomach, were in these cases, as they probably will be in all, sufficient evidence that the disease was chronic, and had only an accidental connexion with the taking of food. Some very interesting remarks on the medico-legal relations of this affection are contained in a paper by Mr. Alfred Taylor, in the Guy's Hospital Reports for 1839.

